# Seasonal Incidence of Community-acquired Pneumonia: A Retrospective Study in a Tertiary Care Hospital in Kathmandu, Nepal

**DOI:** 10.7759/cureus.6417

**Published:** 2019-12-18

**Authors:** Mohammed Ainnul Haque

**Affiliations:** 1 Medicine, Patan Hospital, Kathmandu, NPL

**Keywords:** season, aetiology, nepal, community-acquired pneumonia, acinetobacter calcoaceticus baumanii (acb) complex

## Abstract

Introduction

Community-acquired pneumonia (CAP) is the major cause of death in adult and elderly persons with a variety of presentations. Seasonal variation in the incidence of the disease is essential for clinicians and epidemiologists who deal with such diseases. The study was aimed at analysing the clinical profile and outcomes of community-acquired pneumonia during different seasons of the year in a tertiary care hospital, Manmohan Memorial Teaching Hospital (MMTH), of Kathmandu, Nepal.

Method

The aetiology and clinical profile of 378 patients with CAP who were admitted to MMTH over a period of one year were taken into account in this retrospective cross-sectional hospital-based study. Data were retrieved from the hospital medical records section and the Department of Pathology. All patients with a primary diagnosis of CAP admitted to the hospital were included in the study. Monthly and seasonal trends, aetiology, comorbidities, and mortality rates were analysed.

Results

Of 378 patients with CAP, 160 patients (42.3%) had associated chronic obstructive pulmonary disease (COPD), 92 patients (24.3%) had hypertension (HTN), 59 patients (15.6%) had diabetes, 12 patients (3.1%) had active pulmonary tuberculosis, seven patients (1.85%) had kidney disease, and the remaining 48 patients (12.6%) had only CAP. Seasonal variation of CAP was noted in 131 patients (35%) in the winter, 98 (26%) in autumn, 86 (23%) in spring, and 63 patients (16%) in summer seasons. None of the patients were vaccinated against influenza and pneumococcus. The most common organism isolated in CAP was Acinetobacter calcoaceticus baumannii (ACB) complex (4.7%), which was more distinguished in the winter season. The second most isolated organism was Pseudomonas aeruginosa (2.6%). The most common clinical presentation was fever (63%), followed by cough (47%) and shortness of breath (47%). Sputum culture was found to be positive in 51 cases (13.4%). Among 378 patients, 78 patients (20.6%) received treatment in the Intensive Care Unit (ICU) and the rest of the patients received treatment in the general medical ward. The mortality rate was found to be 6.6%.

Conclusion

An incidence of sputum-positive CAP was found in 51 cases (13.4%). The most common organism was ACB complex, followed by Pseudomonas aeruginosa, which were sensitive to polymyxins. Both of them were predominant in the winter and spring.

## Introduction

Pneumonia is a disease known to mankind since ancient times. It is an acute inflammation of the lung parenchyma which is caused by various infective and non-infective aetiologies. It gives rise to physical and radiological features compatible, such as pulmonary consolidation in a part or parts of one or both lungs [1]. It is one of the major causes of death and morbidity with an incidence of 20% to 30% in developing countries and 3% to 4% in developed countries [2]. It is projected that Nepal, India, Bangladesh accounts for 40% of global acute respiratory infections [2].

Recently, there is an uptrend in hospitalizations for pneumonia in the United States [3] and other European countries [4]. The increase is ascribed to increment in the proportion of the elderly population and ubiquity of comorbid chronic diseases, such as diabetes mellitus, hypertension (HTN), and chronic obstructive pulmonary disease (COPD). The mortality rate was noted to be about 5% to 35%, with a very poor prognosis in elderly people with comorbid chronic diseases [5]. In different parts of the world, Streptococcus pneumoniae is the major aetiological agent of bacterial pneumonia [6-8]. Other causes include Haemophilus influenzae, Staphylococcus aureus, and Gram-negative bacilli Klebsiella pneumoniae and Pseudomonas aeruginosa. The atypical organisms are the Mycoplasma pneumonia, Chlamydia pneumoniae, and Legionella species [6, 9]. Influenza and pneumococcal vaccinations have a huge impact on morbidity and mortality in elderly patients with comorbid diseases [10-11]. However, not much attention is given to influenza vaccination in the Nepalese population.

Despite the high occurrence of community-acquired pneumonia (CAP), the seasonal incidence of the disease and its aetiologies remain undocumented so far and require a comprehensive study [12]. Moreover, only a small number of studies have been done to determine the seasonal variation of pneumonia in our country. Therefore, this study will describe the aetiological profile and seasonal incidence of community-acquired pneumonia in patients in a tertiary care hospital, Manmohan Memorial Teaching Hospital (MMTH), in Kathmandu.

## Materials and methods

A retrospective cross-sectional hospital-based study was carried out between April 14, 2018 and March 13, 2019 (one year) and involved a total number of 1,375 patients. Among them, 378 patients were primarily diagnosed with CAP and were admitted to the medical ward and ICU of the MMTH. Data were retrieved from the hospital medical records section from discharged patient files of those who had been diagnosed with CAP. The study was approved by the Institutional Review Committee of Manmohan Memorial Teaching Hospital (MMTH).

CAP was diagnosed by the presence of acute pulmonary infiltration in a posteroanterior (PA) chest x-ray with at least two of the following symptoms: fever, cough, and purulent sputum. Patients aged between 15 to 95 years were included irrespective of gender, race, religion, or residence. Exclusion criteria included patients with hospital-acquired pneumonia, positive blood tests for human immunodeficiency virus (HIV), lung malignancies, pregnancy, and neutropenia irrespective of the cause. The variables which were associated with CAP were analysed, such as associated comorbidities, smoking, and the outcome of the treatment. Laboratory reports, such as sputum for gram\acid-fast bacilli stain and sputum culture, were also analysed as study variables to determine the aetiology of the disease. Testing the growth of organisms in sputum culture was done using blood or chocolate agar medium. The study population was divided into eight groups utilizing 10-year intervals. The outcome of the diseases were analysed to whether the patient was discharged, left against medical advice (LAMA), referred to another centre, or died during treatment.

Seasonal climate variation data was taken from the Department of Hydrology and Meteorology, Government of Nepal (http://www.dhm.gov.np/climate/). According to it, the mean daily temperature and relative humidity of the study months were included to examine the seasonal variation of the disease. 

For the statistical analysis, Microsoft® Excel (Microsoft® Corp., Redmond, WA) and the IBM Statistical Package for Social Sciences (SPSS), version 20 (IBM SPSS Statistics, Armonk, NY) were used. Descriptive analysis was used, such as mean, the standard deviation for continuous variables, and frequency occurrence. The percentage was used to categorize the variables, and the Chi-square test was used to determine the significance of the difference between the variables. The p-value of < 0.05 was considered significant. 

## Results

**Figure 1:**
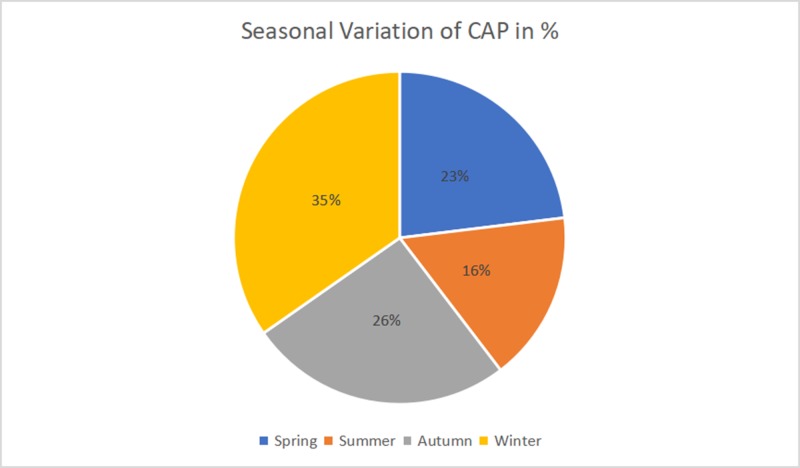
Seasonal distribution of community-acquired pneumonia (CAP) by season

Of the 378 cases of CAP, 173 were male and 205 were female with a female to male ratio of 1.1:1. The mean age was 61.1 years with the standard deviation (SD) of 3.45. Cigarette smoking was noted in 52 patients (13.8%). None of the patients were vaccinated against Hemophilus influenza or pneumococcus. The highest incidence of CAP was noted in the winter season at 36%, followed by autumn (26%) and spring (23%). However, the low incidence of 16% was noted in the summer season, as shown in Figure 1. No significant association were found between sex and seasonal variation (X^2^ = 1.0569, degrees of freedom (df) = 3, p-value = 0.7875).

**Figure 2:**
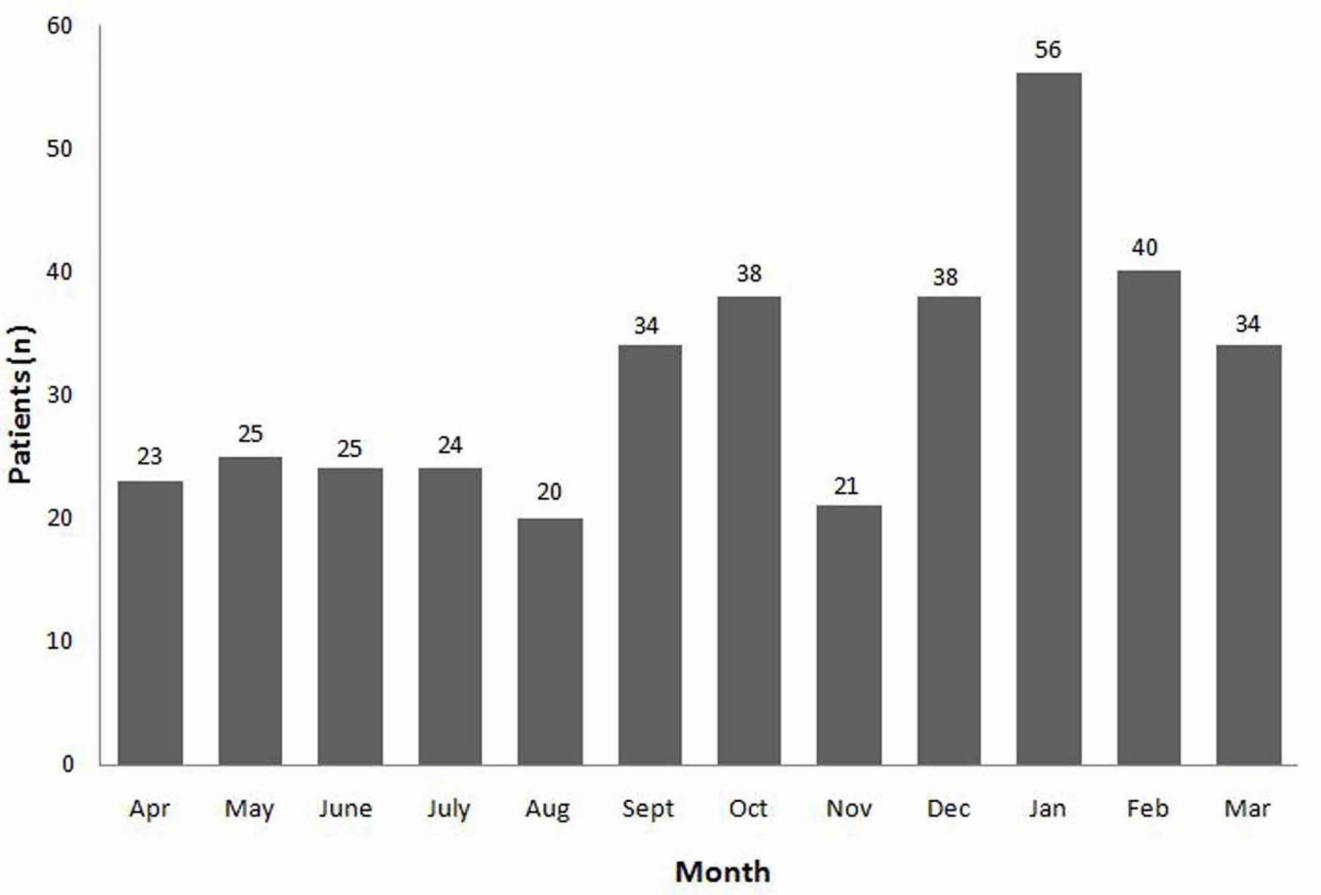
Seasonal distribution of community-acquired pneumonia (CAP) every month of the year studied (2018 - 2019)

To determine the seasonal variation in hospital admissions, mid-March to May is defined as spring, June to August as summer, September to November as autumn, and December to mid-March as winter. The average temperature during the study year ranged from 24.3° C in the summer to 10.9° C in the winter. Table 1 and Figure 2 show the seasonal variation of the study months during April 2018 and March 2019.

**Table 1:** Seasonal Variation of Climate in Kathmandu During April 2018 to March 2019 (One Year)

Month	Mean Daily Temperature ⁰ C	Mean Daily Relative Humidity %
April 2018	20⁰ C	65%
May 2018	22⁰ C	73%
June 2018	24⁰ C	80%
July 2018	24⁰ C	87%
August 2018	24⁰ C	88%
Sept 2018	23⁰ C	82%
Oct 2018	19⁰ C	73%
Nov 2018	14⁰ C	74%
Dec 2018	11⁰ C	73%
Jan 2019	10⁰ C	72%
Feb 2019	12⁰ C	72%
March 2019	16⁰ C	64%

**Figure 3:**
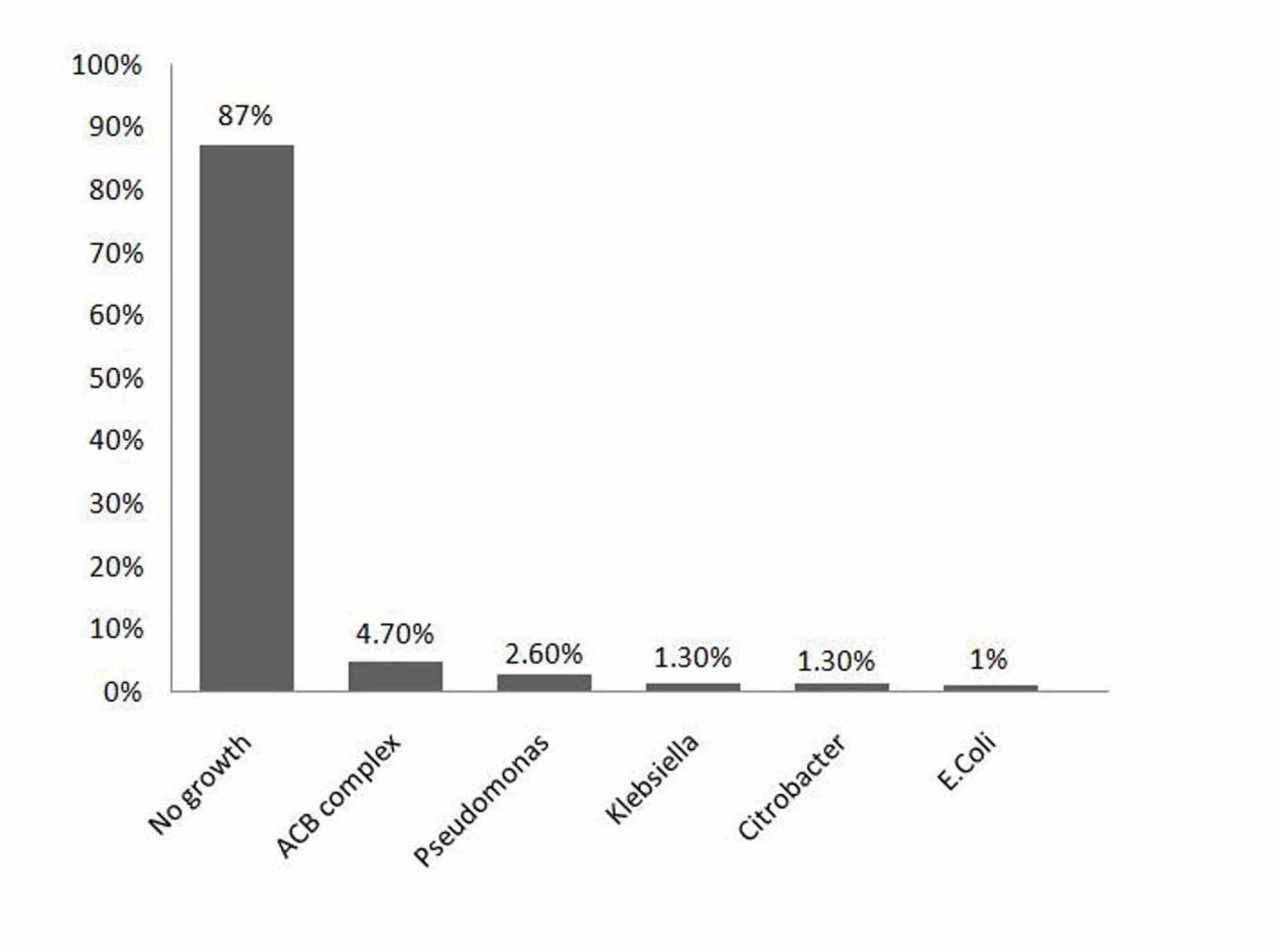
Growth pattern of organisms in sputum culture in patients with community-acquired pneumonia (CAP) ACB: Acinetobacter calcoaceticus-baumannii; E. coli: Escherichia coli

Acinetobacter calcoaceticus baumannii (ACB) was found in 18 patients (4.7%), followed by Pseudomonas aeruginosa which was noted in the sputum cultures of 10 patients (2.6%). ACB was more prevalent in the winter (2.3%), whereas P. aeruginosa (1.3%) was more prevalent in the spring. Tables 2-3 show the frequency distribution and seasonal variation of etiologies. Figure 3 shows the growth pattern of organisms in sputum cultures.

**Table 2:** Frequency Distribution of Community-acquired Pneumonia Etiologies

Pathogen	Patients	
	Number of Patients	% of Patients
Acinetobacter baumannii-calcoaceticus	18	4.7%
Pseudomonas aeruginosa	10	2.6%
Klebsiella pneumoniae	8	2.1%
Citrobacter freundii	5	1.3%
Escherichia coli	4	1.05%
Haemophilus influenzae	3	0.79%
Enterobacter cloacae	2	0.5%
Enterobacter aerogenes	1	0.2%

**Table 3:** Seasonal Variation of Pathogens Isolated in Community-acquired Pneumonia (CAP)

Pathogen	Spring (n = 87) %	Winter (n = 131) %	Summer (n = 63) %	Autumn (n = 97) %
Acinetobacter baumannii-calcoaceticus	2.1	2.3	-	0.2
Pseudomonas aeruginosa	1.3	0.7	0.2	0.2
Klebsiella pneumoniae	0.5	0.2	0.2	1.0
Citrobacter freundii	0.2	0.7	-	0.2
Escherichia coli	1.0	-	-	-
Haemophilus influenzae	0.2	-	0.2	-
Enterobacter cloacae	-	-	-	0.5
Enterobacter aerogenes	0.2	-	-	-

**Figure 4:**
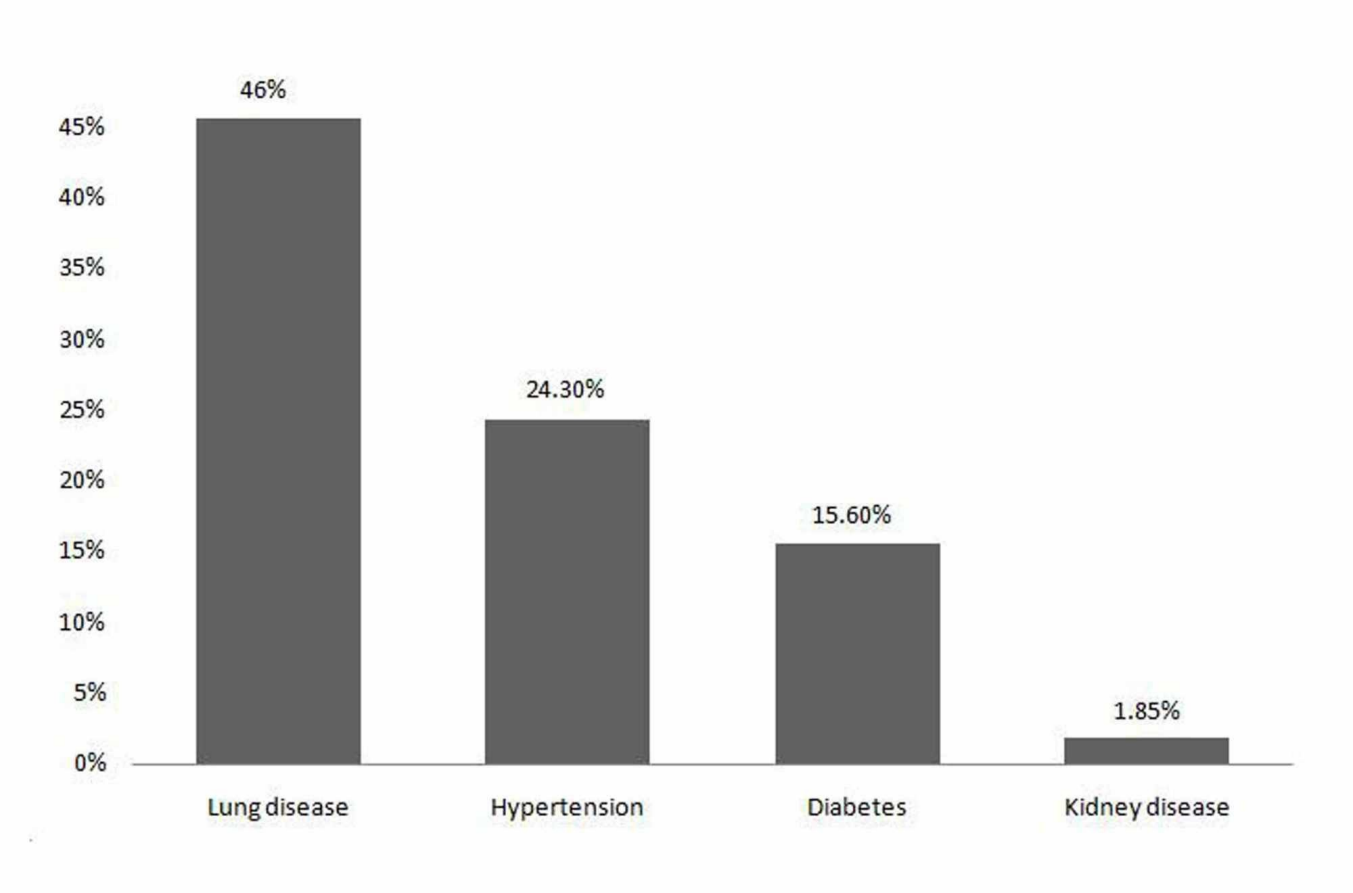
Illustrated comorbid disease associated with community-acquired pneumonia (CAP)

ACB was isolated in 18 cases (n = 18) which were sensitive to polymixins in 12 cases. ACB was also sensitive to ciprofloxacin (five cases), macrolides (four cases), cotrimoxazole (four cases), and aminoglycosides (three cases). Other results of antibiotic sensitivity testing are summarized in Table 4. In the winter season, 131 patients were discharged and 10 patients had expired. Table 5 shows the outcome of hospitalized CAP cases as per the seasonal distribution. There was no significant association between outcomes and seasons (X^2^ = 12.062, df = 9, p-value = 0.2099). Lung disease (46%) was found to be the most common comorbid disease associated with CAP, followed by HTN (34.3%), and diabetes (15.6%) as shown in Figure 4.

**Table 4:** Antibiotic Susceptibility Testing Report

Pathogen	Polymyxins	Ciprofloxacin	Aminoglycoside	Macrolides	Cephalosporin	Cotrimoxazole
Acinetobacter baumannii-calcoaceticus (n = 18)	12	5	3	4	-	4
Pseudomonas (n = 10)	5	5	8	3	3	3
Klebsiella (n = 8)	4	3	5	1	3	3
Citrobacter freundii (n = 5)	3	3	3	1	-	1
Escherichia coli (n = 4)	3	-	4	-	1	-
Haemophilus influenzae (n = 3)	-	-	-	1	-	-
Enterobacter cloacae (n = 2)	2	-	-	-	-	-
Enterobacter aerogenes (n = 1)	1	-	-	-	-	-

**Table 5:** Outcome of Inpatients as Per Seasons LAMA: left against medical advice

Seasons	Outcome of Hospitalized Cases			
	Discharged	Death	LAMA	Referred
Autumn	74	7	8	2
Spring	54	8	4	5
Summer	56	1	5	1
Winter	131	10	5	9

## Discussion

Our study included 378 cases of CAP admitted to the medical ward of one of the teaching hospitals of Kathmandu. The female to male ratio was 1.1:1, which is lower than in a similar study conducted by Shah et al. who found the ratio to be around 1:38 [13]. The mean age was 61.1 years in our study. Other studies also have a similar mean age ranging from 52 - 58 years [13-15]. In our study, fever (63%) was the most common presentation, followed by cough (47%), which is lower than in some studies conducted [13-15]. Breathlessness was present in 47% of the patients in our study which was almost similar to another study [15].

In this study, 63.4% had comorbidities associated with community-acquired pneumonia. A study done by Bansal on CAP showed that 70% - 78% had coexisting comorbidities [15]. The most common comorbidity was COPD which was identical in our study as well. The correlation between comorbidity and the outcome was not significant. It is documented by various literature across the globe that vaccination is beneficial to prevent hospitalization and death [16-17]. In this study sample, no one was found to be vaccinated against H. influenzae or pneumococcus. These findings have clearly illustrated that health care professionals should be more attentive in recognizing and vaccinating the vulnerable population.

The causative agent of community-acquired pneumonia was found in 51 cases (13.4%) in this study. The diagnostic capability is limited in sputum smear microscopy and culture due to a large number of improper collections, a delay in handling, and processing of samples [18]. It is accepted that the application of polymerase chain reaction (PCR) in sputum can enhance the detection of aetiological agents [9, 19]. Some data shows that this technique can confirm aetiological agents in 29% - 75.6% of cases [9, 13, 15]. In our setting, the result was poor due to the lack of facilities performing studies like PCR, detection of pneumococcal antigen in urine samples, and serological tests, such as IgM ELISA, for the confirmation of causative agents. However, this result is even lower than the studies executed in a similar setup like India. This may be due to the unrestrained use of antibiotics without culture sensitivity before the patient receives treatment in a tertiary care hospital.

The most common aetiology of CAP in our study was the ACB complex. ACB is an important nosocomial pathogen. ACB complex is also known for the rapid development of resistance to multiple antibiotics that limits the therapeutic options for the treatment of infections caused. With the emergence of increased resistance even to newer antimicrobials, we are moving towards the “pre-antibiotics era.” A study done in Nepal showed the emergence of multidrug-resistant (MDR) ACB complex [20]. Rampant use of antibiotics, lack of infection control practices (like handwashing), and the absence of surveillance and a reporting system could be some reasons for the problem. Although S. pneumoniae has been documented as the most common causative agent, some other studies have also reported that gram-negative organisms have become a major aetiology these days [14, 21]. Also, in our study, only gram-negative organisms were isolated and they were sensitive to polymyxins, followed by aminoglycosides and ciprofloxacin.

The seasonal incidence of pneumonia was studied over a one-year period in relation to the age, sex, and outcome of the patients. All the patients who were part of this study were hospitalized, and this being a tertiary care hospital, the study covered a huge population from different socioeconomic backgrounds. Mortality from pneumonia is associated with seasonal variation related to outbreaks of influenza and the difference in winter temperature.

The geographical zone where the study was carried out, i.e., Kathmandu, is relatively colder throughout the year. Hospitalization started to increase in December and decrease in February which is a definite winter month in this geographical zone. Plenty of pathophysiological mechanisms have been proposed to illustrate the association of a drop in temperature and acute respiratory infection, such as a reduction in local immune response characterized by decreased phagocytic activity. The major component of the host-specific immune response is the action of phagocytosis in destroying viruses and bacteria is important to prevent infection [22]. Phagocytosis failure is exacerbated by the activation of latent subclinical inflection in the population, resulting in an abrupt increase in the frequency of acute respiratory infections, typically followed by a sudden drop in temperature [23]. Other risk factors that enhance the risk of acute respiratory infections are indoor dwellings with poor ventilation and indoor population.

A study by Fransén reported the number of hospitalizations by months for three years [24]. Peak hospitalization of CAP was seen in the winter and spring seasons. Another study done by Foy et al. over a consecutive five-year period showed three annual peaks that occurred in the spring and two occurred in the winter [25]. However, in our study, it was found that the number of hospitalizations was most marked in the winter instead of the spring which is found to be similar in the study conducted by Cilloniz et al. . Some studies show that the peak incidence was obtained in the winter season which confirms the results of our study . The results yielded showed a greater number of hospital admissions in elderly males aged more than 65 years which is similar to the other studies conducted [26]. In this stage, the elderly population is known to have chronic and weakening conditions and the tendency towards males is high. This fact is reported to be a major risk factor for CAP [27]. As they get older, they are also more vulnerable in developing pneumonia because of the weakened immune system.

## Conclusions

Our retrospective study at MMTH revealed that a high incidence of CAP occurred in the winter season. This suggests winter is proven to be one of the main reasons for the development of community-acquired pneumonia. The admission rate due to CAP was noted to be high in the elderly population, particularly in males above the age of 65 years. Only gram-negative bacteria were isolated in 51 patients (13.4%) and they were sensitive to polymyxins, aminoglycosides, and ciprofloxacin. Surprisingly, our study noted not a single gram-positive bacteria were isolated in the sputum, which is the most common aetiological agent for CAP. In our study, only a few numbers of organisms were isolated in sputum cultures, although they were clinically diagnosed cases of CAP. It may be due to unethical, uncontrolled, and empirical use of antibiotics in the initial stage of the disease. This is still a matter of further research in Nepal.
